# Effect of controlled release of HGF on extracellular vesicle secretion by urine-derived stem cells

**DOI:** 10.3389/fbioe.2024.1436296

**Published:** 2024-08-21

**Authors:** Abdelrahman Alwan, Fatma Khalil, Joshua Bowlby, Gabrielle Peko, Exel Valle Estrada, Sangeeta Singh, Gagan Deep, Yuanyuan Zhang, Alan C. Farney, Emmanuel C. Opara

**Affiliations:** ^1^ Wake Forest Institute for Regenerative Medicine, Wake Forest School of Medicine, Winston-Salem, NC, United States; ^2^ Department of Histology, Faculty of Veterinary Medicine, South Valley University, Qena, Egypt; ^3^ Department of Internal Medicine, Wake Forest School of Medicine, Winston-Salem, NC, United States; ^4^ Department of Surgery, Wake Forest School of Medicine, Winston-Salem, NC, United States; ^5^ Virginia Tech-Wake Forest School of Biomedical Engineering and Sciences, Wake Forest School of Medicine, Winston-Salem, NC, United States

**Keywords:** small extracellular vesicles, Urine-derived stem cells, controlled release, hepatocyte growth factor, alginate microbeads

## Abstract

**Introduction:**

The hepatic growth factor (HGF) stimulates DNA synthesis and cell proliferation and plays a role in tissue protection and regeneration. In this study, we have examined the effect of incubation of HGF with urine-derived stem cells (USCs) on the secretion of small extracellular vesicles (sEV) by the cells.

**Materials and Methods:**

HGF in the incubation medium was either a bolus administration or a controlled release of an equivalent amount from microbeads within the size range of 50–200 µm made with ultrapurified low-viscosity high-guluronic acid (UP-LVG) alginate. USCs were incubated with or without HGF for 3 days or 7 days before removal of the incubation media, followed by harvesting sEV by the precipitation method. The protein content of isolated sEV was measured by bicinchoninic acid assay (BCA) for these three groups: control (no HGF beads), bolus HGF, and HGF beads. We also performed nanoparticle tracking analysis (NTA), Western blot assay, and ELISA for the HGF content of samples.

**Results:**

We found a significantly higher concentration of proteins in the HGF microbead group (control release group) compared to the bolus group and the control group after 7 days (*p* < 0.0017). The NTA data aligned with the BCA; they showed a significantly higher concentration of particles within the size range of sEV (<200 nm) in the group treated with HGF beads compared to the two other groups on day 7 (*p* < 0.0001).

**Conclusion:**

We found that administration of HGF to USCs by controlled release of the growth factor significantly enhances the levels of sEV secretion during 7 days of incubation.

## 1 Introduction

Stem cells have great potential in many scientific and medical fields. Their remarkable capacity to transform into any cell type and the ability to renew themselves have created a great deal of excitement in the scientific community (Martin, 1981; Ding et al., 2011; Zakrzewski et al., 2019). They can be classified into embryonic stem cells, derived from early-stage embryos, and adult stem cells, found in mature tissues, according to their developmental stage (Evans and Kaufman, 1981). Research and clinical trials have demonstrated the effectiveness of both local and systemic stem cell therapies (Gill et al., 2018; Knoll et al., 2024). The results vary depending on whether stem cells have a therapeutic effect by transforming into permanent, functional tissues or if they provide benefits through a temporary presence and the release of regenerative factors (Gill et al., 2018). An emerging strategy in therapy in recent years is the use of small extracellular vesicles (sEV) released by stem cells rather than the cells themselves (Wang et al., 2023), as sEV secreted by stem cells have been shown to have stem cell-like functions, effectively prevent tissue damage, and repair impaired tissues (Wang et al., 2023).

Urine-derived stem cells (USCs) constitute a type of adult stem cells that have many advantages over other types of stem cells, such as skeletal muscle-derived stem cells and adipose-derived stem cells (ASCs), including noninvasive collection, abundant supply, multipotent differentiation potential, immunomodulatory properties, and low risk of tumorigenesis (Yu et al., 2023b). Extracellular vesicles (EV) secreted from USCs have shown multifunctional differentiation potential in *in vitro* studies and have promising applications in different regenerative medicine settings. They have been shown to suppress osteolysis and promote joint osteogenesis (Li et al., 2021a; Li et al., 2021b). Other studies showed EV harvested from USCs could enhance proliferation and migration ability and inhibit apoptosis in knee osteoarthritis (Liu et al., 2022). Intravenous injections of USCs EV showed a potential effect on the kidneys of diabetic rats through decreasing urinary microalbumin excretion and preventing podocyte and tubular epithelial cell apoptosis (Jiang et al., 2016), while local injection of USCs EV showed significant improvement in stress urinary incontinence (SUI) in rat models (Wu et al., 2019). The focus was on the sEV rather than other extracellular vesicles secreted by cells, as, in addition to their low immunogenicity, the small size of the sEV was considered the perfect cargo for drug delivery as it allows a higher cellular uptake than larger-size EV, as seen in several studies (Hoshyar et al., 2016; Caponnetto et al., 2017; Yeh et al., 2020). It is known that the hepatic growth factor (HGF) has anti-inflammatory effects, which probably explains its antifibrotic role in glomerulosclerosis (Esposito et al., 2005). In addition, a recent study has shown that adipose-derived MSCs overexpressing HGF secrete exosomes that have therapeutic effects on liver injury (Yu et al., 2023a).

Studies have shown that the composition of the sEV cargo plays a crucial role in determining the ultimate outcome of the interactions of sEV with various cell types (Zeng et al., 2023). However, loading cargo into sEV remains a challenge. Despite various strategies such as incubation, electroporation, sonication, extrusion, freeze–thaw cycling, and transfection developed to facilitate cargo loading, inadequate efficiency persists (Zeng et al., 2023). Although previous studies have shown that adiponectin stimulates exosome release to enhance mesenchymal stem cell (MSCs)-driven therapy (Nakamura et al., 2020; Kita and Shimomura, 2021), it is presently not known if HGF can stimulate sEV secretion in cells. Therefore, the purpose of the present study was to determine the effect of incubation of USCs with HGF either as a bolus or in a controlled release manner on sEV secreted by the cells.

## 2 Materials and Methods

UP-LVG was purchased from Nova-Matrix and was reported by the manufacturer to have a molecular weight of 75–200 kDa and a viscosity of 20–200 mPa s (Cat # 4200001). HGF was purchased from PeproTech (Cat # 315-23), and the HGF ELISA kit was obtained from RayBiotech (Cat # ELH-HGF). A bicinchoninic acid assay (BCA) kit was purchased from Thermofisher Scientific (Cat # 23227). CD9 and TSG101 Western blot antibodies were purchased from Abcam (ab275018), and secondary Ab, Anti-mouse, and Anti-rabbit IgG were purchased from Thermofisher (Cat # A16066 & 65–6120), respectively. Other Western blot reagents were purchased from Bio-Rad. Running buffer (Cat # 1610772), transfer buffer (Cat # 1610771), 2-mercaptoethanol (Cat # 1610710), 4X Laemmli sample buffer (Cat #1610747), and blotting grade blocker (Cat # 1706404). All other materials and media were purchased from Sigma-Aldrich unless otherwise stated.

### 2.1 USCs isolation, characterization, and complete culture medium preparation

#### 2.1.1 USCs isolation

For urine sample collection: donors were male patients within the age range of 28–46 years, and the collection was conducted in a clean room, in a sterile container. Before collecting urine, the penis glans was wiped with an alcohol napkin three times. After mid- and last-stream urine was collected, the neck of the collecting container was wiped with an alcohol napkin. To increase the survival rate of the USCs, DMEM containing 10% fetal bovine serum (FBS) was added to the container before collecting the sample. The isolation process started within 2 hours after collection.

To isolate the USCs, the urine sample was poured into 50-mL tubes and washed via centrifugation (5 min, 1500 RPM). After washing, the supernatant was carefully collected, leaving 1–2 mL supernatant containing the precipitate in the bottom of each tube.

The precipitate was resuspended in 5 mL of Dulbecco’s phosphate-buffered saline (DPBS) in each tube, and the suspensions from all tubes were pooled. DPBS was added to the tubes containing all precipitates to increase the volume to 50 mL. The cells were washed via centrifugation for the second time using the same settings. The supernatant was carefully collected, leaving 1 mL of the supernatant with the precipitate. Then, the cells were resuspended in USCs complete media, counted, and the suspension was poured into a 24-well plate (500 cells per well) (Supplementary Figure S1).

#### 2.1.2 USCs characterization

USCs were characterized as previously described, using immunofluorescence, Western blot, and flow cytometry (Zhang et al., 2008; Lang et al., 2013; Shi et al., 2022). USCs were shown to express the urothelial cell markers, that is, uroplakin Ia and CKs 7, 13, 17, and 19, which are similar to the cultured urothelium obtained from bladder tissue biopsies. Western blot findings confirmed that uroplakin Ia and CK 13 were present in urine-derived cells.

#### 2.1.3 Culture medium preparation

A 500-mL aliquot of keratinocyte serum-free medium (Gibco™ 17005042) was supplemented with FBS (50 mL), Dulbecco’s modified Eagle medium/nutrient mixture F-12 (DMEM-F12 125 mL), DMEM high glucose (375 mL), cholera toxin (30 ng/mL 0.5 mL), 3,39,5-triiodo-L-thyronine (0.5 m)L, 1% penicillin–streptomycin (10 mL), 0.4 g/mL hydrocortisone (0.5 mL), and adenine (1 mL).

### 2.2 Alginate microbead preparation

UP-LVG (1.5% w/v) was dissolved in 50 mM calcium–ethylenediaminetetraacetic acid (Ca–EDTA) pH = 8 by mixing overnight at 4°C. Alginate beads were generated using a microfluidic chip device, as previously described (Enck et al., 2020). Briefly, droplets of the alginate solution were generated by mixing with 1% Span in sterile mineral oil (Sigma-Aldrich Cat #M5310-1L), and alginate droplets were crosslinked by mixing with a solution of 3% acetic acid in sterile mineral oil. The beads were collected in a 50-mL conical tube. Excess oil was aspirated from the solution before washing the beads with hexane three times, letting the beads settle each time before aspirating, and fresh hexane was added to the microbead pellet. The hexane dissolves the residual mineral oil, allowing for effective transfer from the organic phase to aqueous media. The beads were finally washed with 100 mM calcium chloride (CaCl_2_) solution three times by suspending the beads in 100 mM CaCl_2_ for 5 min, followed by centrifugation at 800 RPM for 4 min. The supernatant was discarded before fresh CaCl_2_ was introduced. After the last wash, the beads (Supplementary Figure S2) were collected in micro-Eppendorf tubes for downstream analysis and studies. We studied three groups of USCs incubations, namely: HGF-loaded beads incubated with USCs (HGF beads), empty beads incubated with USCs (Control), and USCs with HGF added directly to the complete cell culture media (Bolus group).

#### 2.2.1 HGF bead and empty bead preparation

In the empty bead group, the beads were added directly to the complete media. In the HGF bead group, the prepared alginate beads were soaked in 2 ng/mL HGF solution while rotating overnight at 4°C. After 24 h, the beads were washed once with 100 mM CaCl_2_ and added to the complete media with USCs.

#### 2.2.2 Bolus group preparation

**FIGURE 1:**
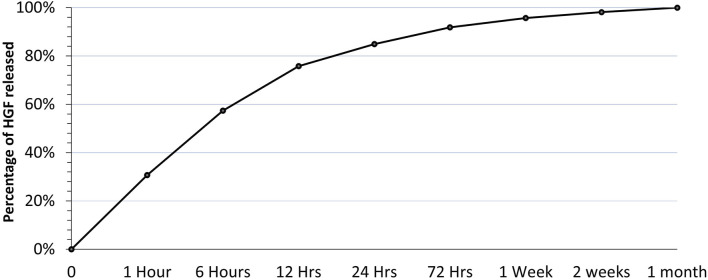
Showing the release profile of HGF from HGF beads over 1 month.

We studied the encapsulation efficiency and the release profile of HGF from the HGF-loaded beads (Figure 1) to determine the appropriate concentration of HGF to be added to the bolus group because it had to match the amount released from the beads in the HGF bead group over the first week.

HGF-loaded beads were prepared as described above and incubated in 25 mM CaCl_2_ in 0.9% sodium chloride (NaCl). Samples from the soaking solution were collected over a duration of 1 month and at set time points; the HGF levels in these samples were measured using ELISA at the end of 1 month. During the ELISA, alginate beads from the incubations were dissolved using 50 mM sodium citrate, and the levels of HGF remaining inside the beads were measured using the same ELISA. Then, the calculated amount of HGF released in the first week was added to the complete media in the bolus group for the 3-day and 7-day incubations of USCs with HGF.

### 2.3 USCs culture

Twelve 60 mm culture dishes were divided into two groups: a day 3 group and day 7 group, and each group was further divided into three subgroups: an HGF bead group, an Empty bead group, and a Bolus group (Supplementary Figure S3). In each dish, 300,000 USCs were added with 4 mL of complete culture media and incubated at 37°C under a 5% CO_2_-humidified atmosphere for 3 days. After 3 days, for the day 3 group, the medium was replaced with 4 mL of USCs media prepared without FBS (serum-free media) and incubated for another 2 days before the cell culture supernatant was collected (Supplementary Figure S4A). For the day 7 group, only 2 mL of the media was replaced with fresh 2 mL of USCs complete culture media for another 4 days after the first 3 days of incubation. After the additional 4 days, the media was replaced with 4 mL of serum-free media and incubated for another 2 days before the cell culture supernatant was collected (Supplementary Figure S4B).

### 2.4 sEV isolation

For sEV isolation, the cell culture supernatant from each group was collected and centrifuged for 30 min at 2000× g to eliminate the cell debris. Following centrifugation, the supernatant was collected, and the total exosome isolation reagent (ThermoFischer Cat # 4478359) was added to the collected media according to the manufacturer’s instructions and incubated at 4°C overnight. After incubation, the solution was centrifuged for 1 h at 10,000× g at 4°C. Following centrifugation, the supernatant was discarded, and the sEV pellet was resuspended in DPBS. The resuspended sEV pellet was stored at −20°C for downstream analysis.

### 2.5 Bicinchoninic acid assay (BCA)

The BCA assay is among the most frequently used methodologies for protein determination, and it is notable for its simplicity, sensitivity, repeatability, and reproducibility (Cortés-Ríos et al., 2020). It provides an accurate determination of protein concentration with most sample types. Therefore, we used the BCA assay to measure the protein concentration in sEV pellets obtained from different groups following the manufacturer’s protocol. This protein concentration was also used to measure samples for nanoparticle tracking analysis along with the Western blot.

### 2.6 Nanoparticle tracking analysis (NTA)

NTA is a rapid and highly sensitive method for the visualization and characterization of EV (Comfort et al., 2021). This technique combines laser light scattering microscopy and Brownian motion and then relates the movement to a particle size, thus determining the size distribution of nanoparticles in liquid suspensions (Filipe et al., 2010; Saveyn et al., 2010; Yang et al., 2014; Kim et al., 2019). Briefly, a Nanosight NS300 (Malvern Instruments, UK; Software Version 3.4.4) was used to measure the size distribution and concentration (particles/mL) to assess the particles within the size range of sEV (<200 nm) in the EV pellet obtained from different groups. We recorded five videos of 30 s for each sample, and the average of five videos was presented as the final size and concentration. The NS300 was calibrated using polystyrene beads of known sizes (100 and 200 nm).

### 2.7 Western blot

To confirm the presence of sEV in the isolated pellet from the cell incubation media, the Western blot assay was performed for the following sEV markers: TSG101, syntenin-1, ALIX, and CD9. Briefly, frozen samples were placed in an ice bath to slowly thaw. A 15% running gel of sodium dodecyl sulfate-polyacrylamide gel electrophoresis (SDS-PAGE) was solidified, and then a 5% stacking SDS-PAGE gel was placed above and allowed to set according to Bio-Rad recommendations. A 3-μg sample of sEV was mixed with a 4x Laemmli sample buffer and 2-mercaptoethanol to dissociate the disulfide bonds, incubated at 95°C for 5 min, and placed back into the ice bath. The positive control and the experimental samples were loaded into the corresponding wells and run at 80 V for 30 min and then at 120 V for 90 min. They were then loaded onto the Bio-Rad semi-dry blotting instrument and run at 12 V for 90 min to transfer samples from the SDS-PAGE gel to the polyvinylidene fluoride (PVDF) membrane. PVDF membranes were placed in opaque boxes and blocked with 5% skim milk overnight, then washed four times with 1xTris buffered saline (TBS) before 1:1000 primary antibody in 1% skim milk was added, and incubated overnight. Next, the primary antibody was washed five times with 1x TBS. A 1:5000 secondary antibody in 1% skim milk was added, incubated overnight, and then washed four times with 1x TBS. Finally, the membrane was read on the iBright imaging system.

### 2.8 Transmission electron microscopy (TEM)

Extracellular vesicle suspensions in PBS from different groups were aliquoted with 15 uL of 2% paraformaldehyde. Formvar/carbon 200-mesh copper grids (Ted Pella, Inc., Redding, California) were washed with ethanol for 1 min and wicked dry with the absorbent paper. Samples were then incubated on the coated side of the grid for 5–30 min. The samples were wicked dry with absorbent paper again and then stained with 1% uranyl acetate for 1 min. The uranyl acetate was removed by wicking on absorbent paper and then placed into the Tecnai BioTwin Transmission Electron Microscope (FEI, Hillsboro, OR). Images were taken at two different magnifications ×49,000 and ×98,000.

### 2.9 HGF ELISA

HGF ELISA was used according to the manufacturer’s instructions to measure the concentration of HGF in samples obtained from different time points in the release study and to measure the concentration of HGF remaining inside the beads at the end of the release study. It was also used to measure the concentration of HGF in the EV pellets obtained from the different experimental groups.

### 2.10 Statistical analysis

Statistics evaluation of data was performed using Prism software, and significance was established as a *p*-value of <0.05. The significances between groups of cell incubations were compared using 2-way ANOVA followed by a Tukey’s post-hoc multiple comparison test.

## 3 Results

### 3.1 Bicinchoninic acid assay

**FIGURE 2:**
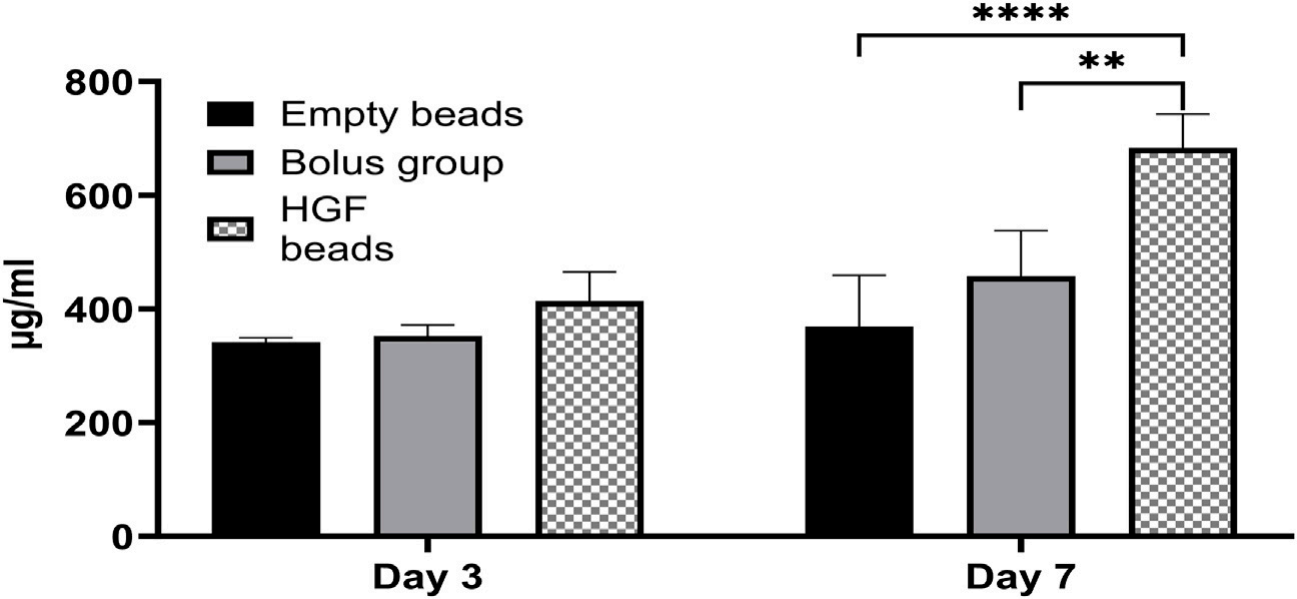
BCA assay showing the concentration of proteins in EV pellets obtained from different groups at day 3 and day 7 (2-way ANOVA **p < 0.0017, ****p < 0.0001).

There was no significant difference in the protein concentrations in the EV pellets obtained from different groups on day 3. In contrast, on day 7, there was a significantly higher protein concentration in the EV pellet obtained from the HGF bead group than those from the bolus group and empty bead control, indicating a superior effect of sustained delivery of smaller amounts over bolus delivery of a high HGF concentration (Figure 2).

### 3.2 Western blot

All groups presented with positive EV marker bands at 24 kd (CD9) and 44 kd (TSG 101) with thicker bands at day 7, indicating increased protein concentration with longer incubation (Supplementary Figure S5).

### 3.3 Nanoparticle tracking analysis

**FIGURE 3:**
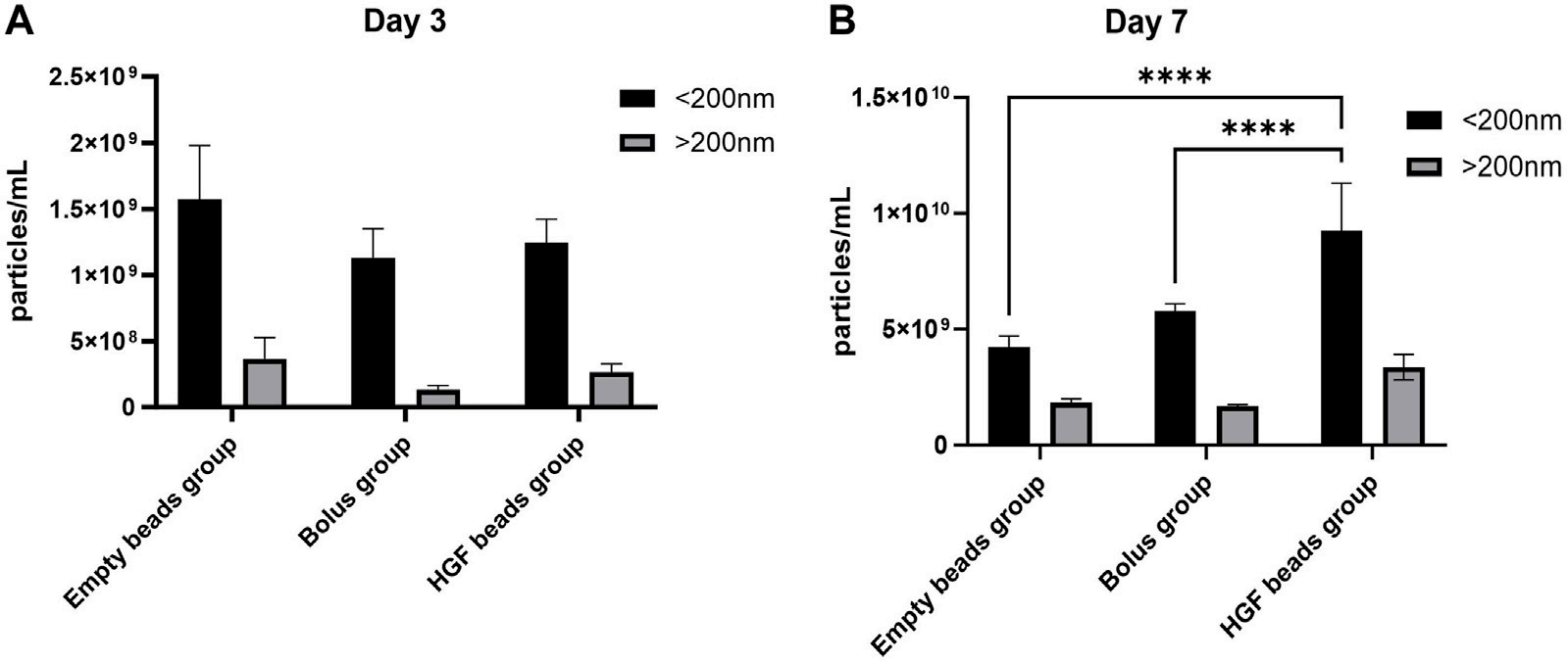
NTA analysis for an sEV pellet obtained from the day 3 group **(A)** and an sEV pellet obtained from the day 7 group **(B)** (2-way ANOVA, ****p < 0.0001).

Most of the particles in the EV pellets (85% of the day 3 group and 73% of the day 7 group) were in the size range of sEV (<200 nm). Day 7 showed higher numbers of sEV-sized particles in the HGF group and the bolus group *versus* the empty bead group, confirming not only the stimulatory role of HGF on sEV production but also the relatively better delivery of the HGF through controlled release *versus* the bolus delivery (Figure 3). In the present study, we were interested only in the sEV and, therefore, did not analyze the changes in EV larger than 200 nm.

### 3.4 Transmission electron microscopy (TEM)

TEM was utilized to confirm the presence of sEV. Spherical sEV were present within a size range of less than 100 nm. This provides evidence of the presence of sphere-shaped vesicles with a lipid bilayer within the size range of exosomes (Supplementary Figure S6).

### 3.5 HGF ELISA

#### 3.5.1 HGF concentrations in the release study samples

**FIGURE 4:**
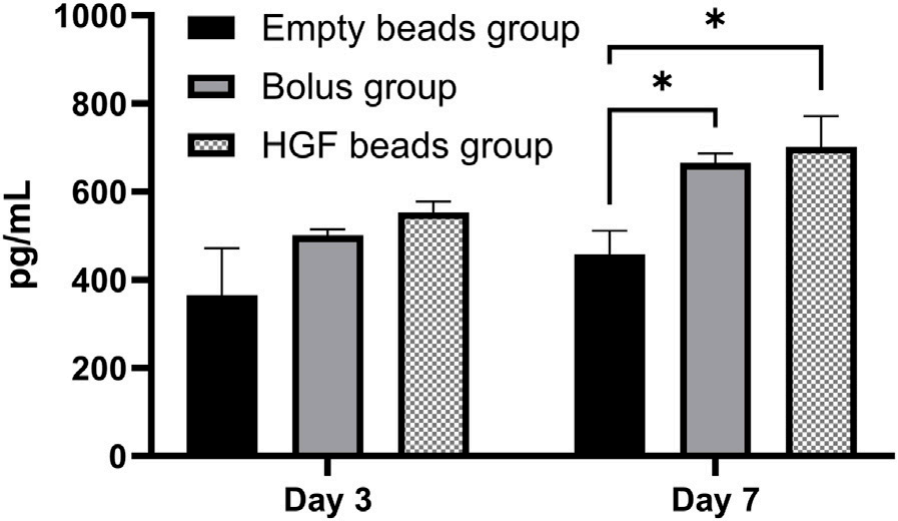
HGF ELISA showing the concentration of HGF in sEV pellets obtained from different groups on day 3 and day 7 (2-way ANOVA *p < 0.0173)

The concentration of HGF released was measured at different time points. The first time point (1 h) showed the highest amount of HGF released, representing a burst release of HGF from the beads. After the first time point, HGF release gradually diminished over time, reaching a plateau by the end of the first month (Figure 4).

After adding up all the HGF released from the beads and the HGF remaining inside the beads at the end of the first month, The encapsulation efficiency was calculated to be 37%; that is, 37% of the initial amount of HGF was absorbed inside the alginate beads after 24 h of soaking. Based on this encapsulation efficiency and the release profile, the bolus dose of HGF was calculated to be equivalent to the total amount of HGF released from the beads at the end of 1 week.

#### 3.5.2 HGF concentrations in the sEV pellet

The HGF concentrations in the different sEV pellets were measured using the same HGF ELISA, and the results show a significantly higher concentration of HGF in the sEV pellets obtained from the HGF bead and bolus groups *versus* the empty bead group only on day 7. However, there was no statistically significant difference between the bolus and the HGF bead groups, demonstrating that the amounts of HGF in both incubations were similar (Figure 4).

## 4 Discussion

Previous studies have shown superior performance of controlled delivery of FGF-1 in increasing vascular density when compared to bolus administration. Thus, a report (Moya et al., 2009) described how microbeads loaded with FGF-1 (total amount 150 ng) were implanted into a surgically created omentum pouch in rats and were compared to control empty microbead implants and a single bolus injection of 150 ng of FGF-1 with an empty microbead implant. Animals were sacrificed at either 3 or 6 weeks post implantation, and omental samples were analyzed for vascular density and mural cell interactions. The vascular area for bolus FGF-1 and FGF-1 loaded microbeads was higher than the control at 3 weeks. At 6 weeks, the vascular density in the group with FGF-1-loaded microbeads was significantly higher than the group with a bolus administration of FGF-1 (Moya et al., 2009).

Therefore, we hypothesized that administering HGF in a controlled release manner rather than bolus delivery would lead to significantly higher stimulation of sEV secretion by USCs. It is interesting that the data generated in the present study *in vitro* study follow the same pattern of effects as the prior *in vivo* study when controlled release delivery is compared with bolus administration of a growth factor. We found significant differences between the levels of sEV concentration (particles/mL) and cargo (protein concentration) on day 7 but not on day 3 in our present *in vitro* studies, similar to the pattern in the *in vivo* study showing a difference between short-term and long-term exposure to a growth factor *in vivo* (Moya et al., 2009). Alginate beads were used to encapsulate the growth factors in both studies. Alginates are a class of biodegradable polymers, used particularly for hydrogels designed for controlled molecular delivery, which can serve as advantageous carriers to support tissue regeneration and healing (Camarata et al., 1992). Alginate, as a biomaterial, has found extensive applications in the realms of biomedical science and engineering, primarily owing to its beneficial properties such as biocompatibility, easy gelation, and tunable microbead characteristics (de Vos et al., 2006; Lee and Mooney, 2012).

USCs are epithelial cells of kidney origin that are obtained non-invasively and possess high stemness properties, self-renewal ability, trophic effects, multipotent differentiation potential, and immunomodulatory ability. These cells show versatile potential for tissue regeneration, with extensive evidence supporting their use in the repair of epidermal and urothelial injuries (Yin et al., 2022). Interestingly, recent studies have indicated that implanted cells do not survive for long and that the benefits of MSCs therapy may be attributable to the vast array of bioactive factors that they produce, which play an important role in the regulation of key biological processes. Thus, secretome derivatives, such as conditioned media or sEV, may present considerable advantages over cells for manufacturing, storage, handling, and product shelf life. They have potential as a ready-to-use off-the-shelf biologic product, albeit regulatory requirements for manufacturing and quality control will be necessary to establish the safety and efficacy profile of these products (Vizoso et al., 2017).

In the present study, we have shown that controlled release of HGF to USCs during 7-day *in vitro* incubation enhances the secretion of sEV by these kidney epithelial cells. It has also been shown that human biliary epithelial cells (BECs) proliferate in response to human hepatocyte growth factor (hHGF) and retain the BEC-specific phenotype, and primary BEC isolates demonstrated dose-dependent proliferation in response to hHGF with a clear increase in cell number, resulting in near-confluent monolayers within 7–10 days (Joplin et al., 1992). Therefore, it appears that a minimum of 7 days may be required for HGF to enhance the cell numbers that caused the increased secretion of the levels of sEV proteins seen on day 7 but not day 3 in the present study. Joplin et al. also reported that BECs showed dose-dependent growth in response to 0.01–100 ng/mL bHGF. The maximum S-phase labeling index reached 40% with half-maximal stimulation at 1 ng/mL (Joplin et al., 1992). Thus, the controlled release approach for delivery of the growth factor to the USCs would provide an enabling environment with smaller doses to achieve the maximum growth rate in contrast to the bolus administration of a single high-dose HGF.

## 5 Conclusion

Various clinical trials have utilized EV secreted by MSCs isolated from various tissues, primarily as a substitute for mesenchymal stem cell therapy. Both pre-clinical and clinical studies indicate that sEV released by the stem cells could potentially replicate some therapeutic benefits of the donor cells while avoiding the inherent limitations of stem cell therapy. Thus, interest in using sEV for regenerative medicine treatments is growing, but generating sufficient levels of sEV for therapeutic purposes remains a challenge. Our present study, which shows the use of controlled release of HGF to enhance the level of sEV secretion in cultured USCs, provides a good strategy to address this challenge.

## Data Availability

The original contributions presented in the study are included in the article/Supplementary Material; further inquiries can be directed to the corresponding author.

## Supplementary material

SUPPLEMENTARY FIGURE S1 Diagram to illustrate urine processing, USCs isolation, and USCs culture.

SUPPLEMENTARY FIGURE S2 Alginate beads prepared using a microfluidic device with a size range of 50–200 µm.

SUPPLEMENTARY FIGURE S3 10× magnification of different cell culture groups: HGF bead group **(A)**, empty bead group **(B)**, and Bolus group **(C)**.

SUPPLEMENTARY FIGURE S4
**(A)** The plan for day 3 groups describes a media change on day 3 to serum-free media and collection of the cell culture supernatant after 2 days (day 5) for sEV isolation. **(B)** The plan for day 7 groups describes a media change on day 3 with complete media, a media change on day 7 with serum-free media, and cell culture supernatant collection after 2 days (day 9) for sEV isolation.

SUPPLEMENTARY FIGURE S5 Western blot analysis of the sEV markers showing positive anti TSG101 **(A)** and positive anti CD-9 **(B)**, (D7 = Day 7; D3 = Day 3).

SUPPLEMENTARY FIGURE S6 Transmission electron microscopy confirms the presence of sphere-shaped vesicles within the size range of exosomes.

## Abbreviations

ADSC-EV, adipocyte derived stem cells extracellular vesicles; ADSCs, adipose-derived stem cells; EV, extracellular vesicles; FBS, fetal bovine serum; HGF, hepatocyte growth factor; MSCs, mesenchymal stem cell; sEV, small extracellular vesicles; UP-LVG, ultrapurified low viscosity guluronic acid alginate; USCs, Urine-derived stem cells.
