# Supplementary figures and images for: Effect of controlled release of HGF on extracellular vesicle secretion by urine-derived stem cells
Source: Front Bioeng Biotechnol. 2024 Aug 21;12:1436296. doi: 10.3389/fbioe.2024.1436296 (PMC11371732; doi:10.3389/fbioe.2024.1436296)

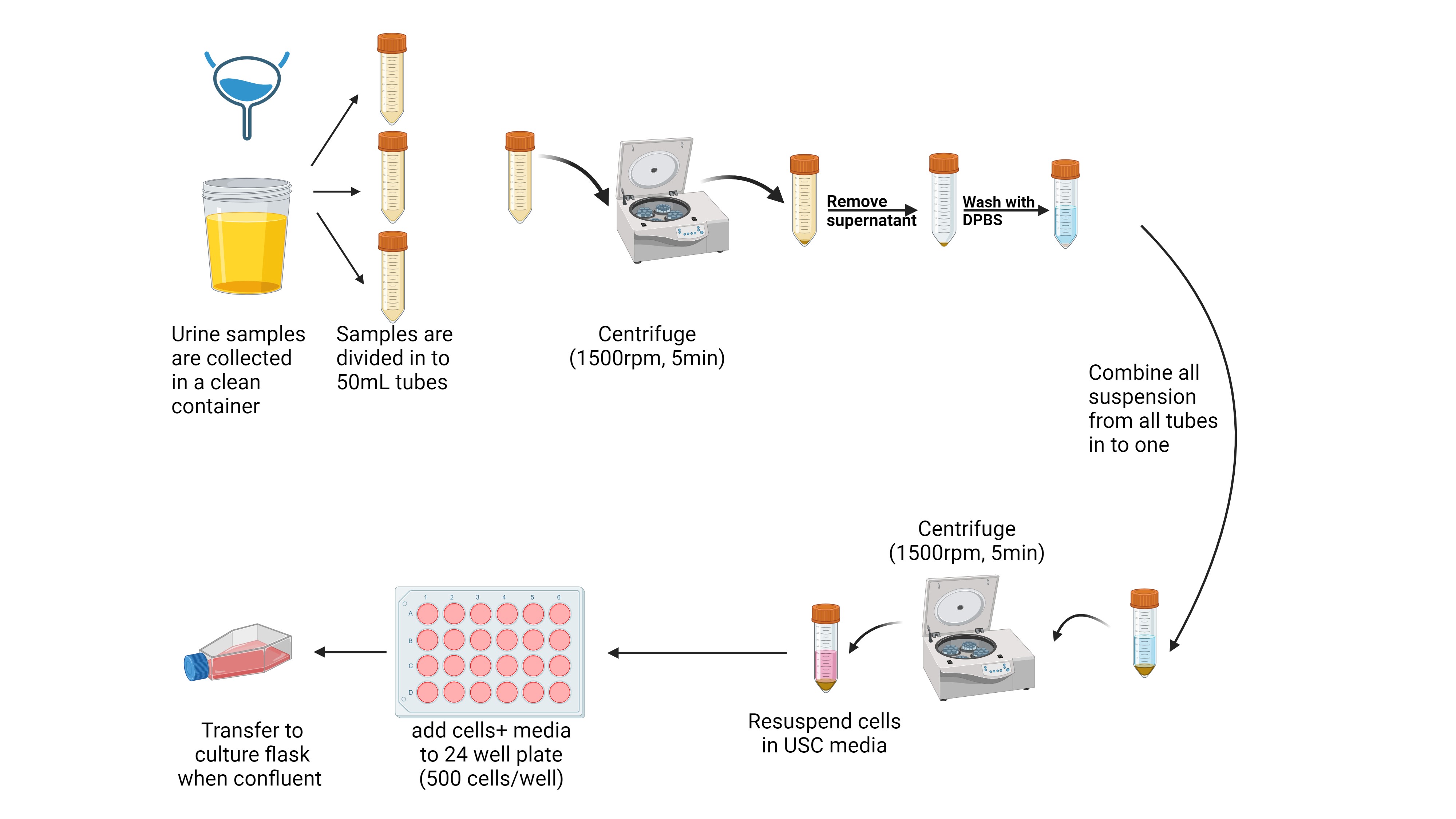

Supplement: Supplementary file 1 [file DataSheet1.zip › Figure S1.JPEG]

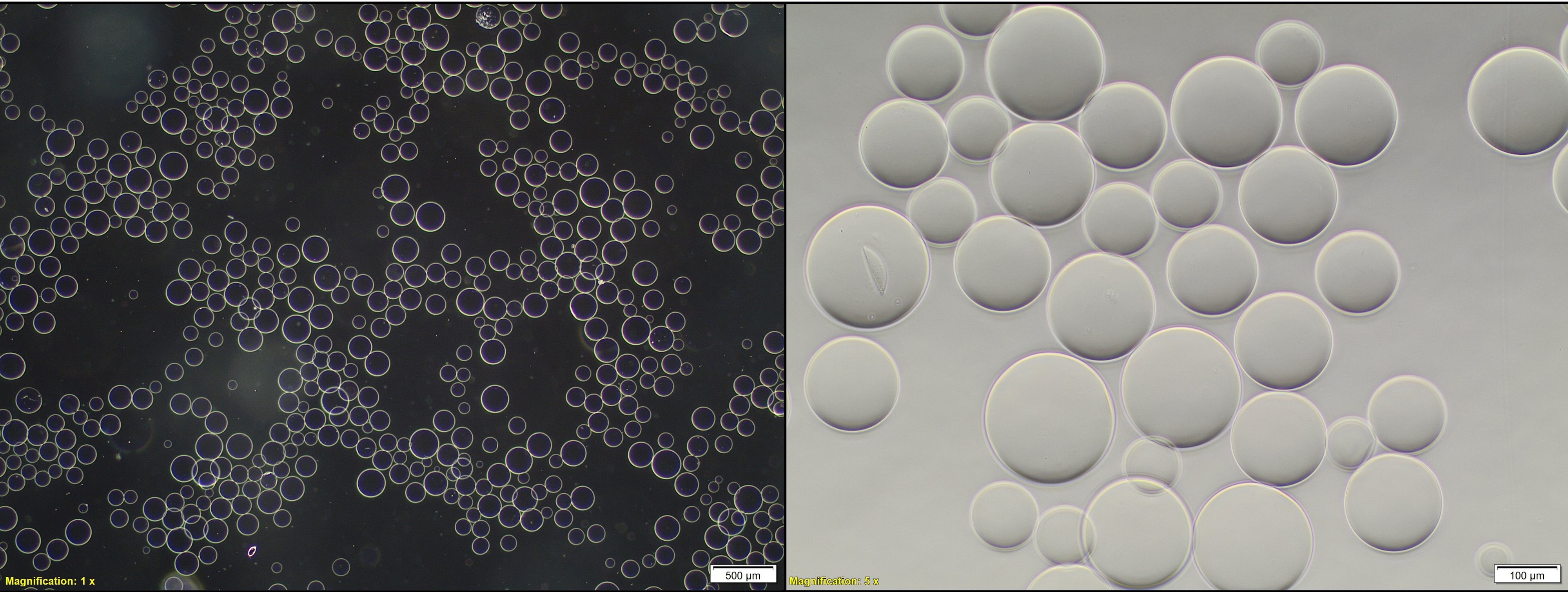

Supplement: Supplementary file 1 [file DataSheet1.zip › Figure S2.JPEG]

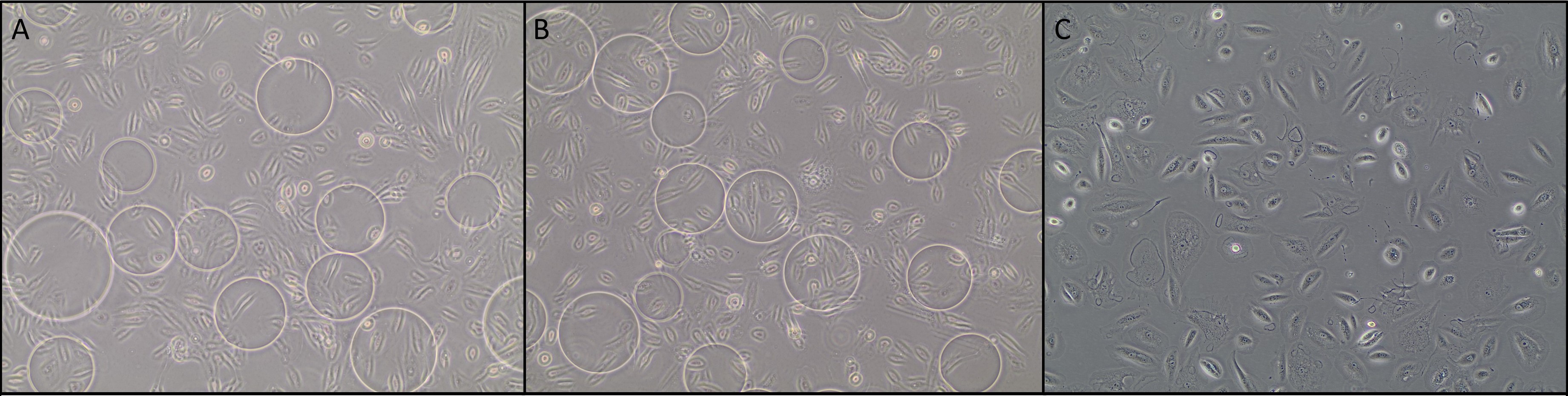

Supplement: Supplementary file 1 [file DataSheet1.zip › Figure S3.JPEG]

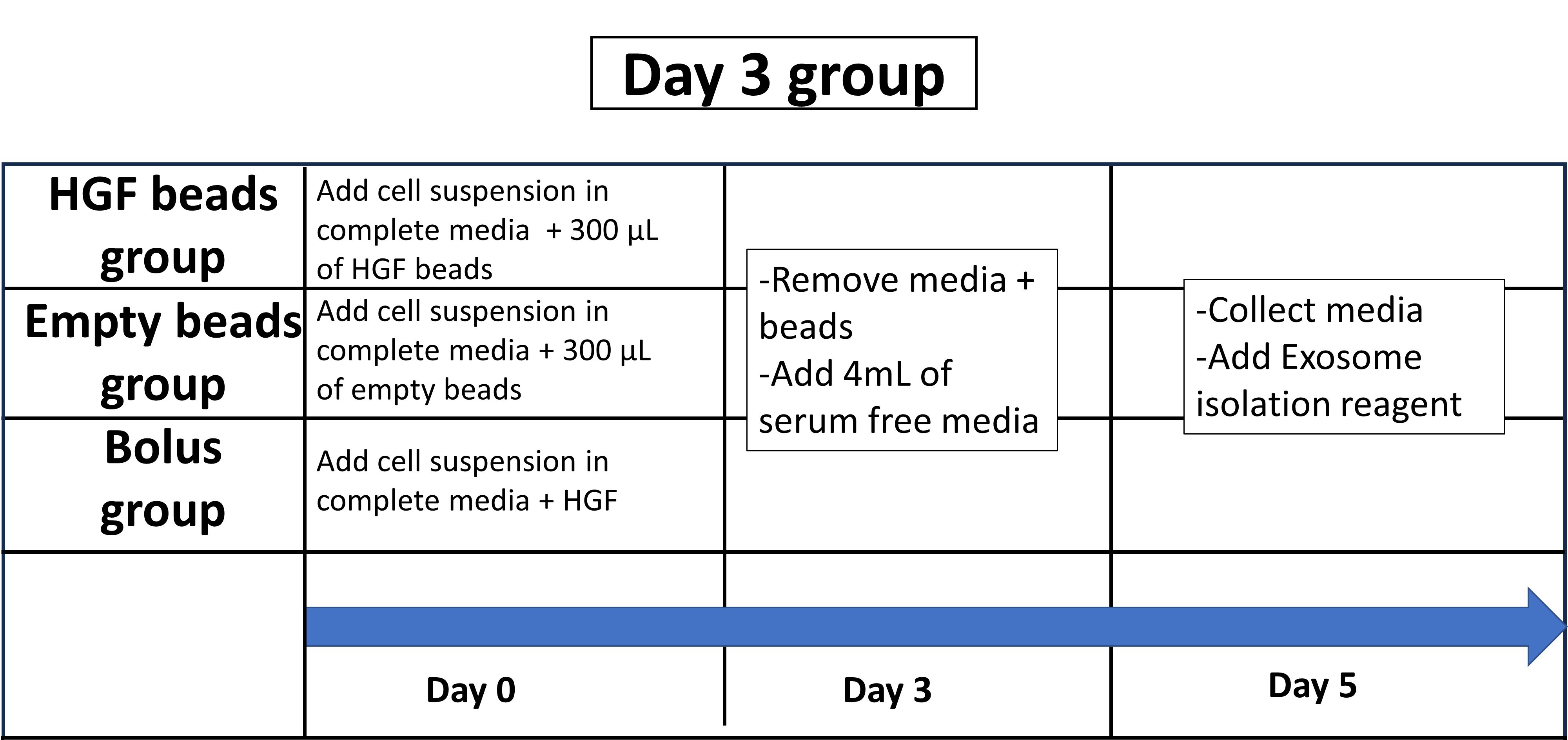

Supplement: Supplementary file 1 [file DataSheet1.zip › Figure S4 A.JPEG]

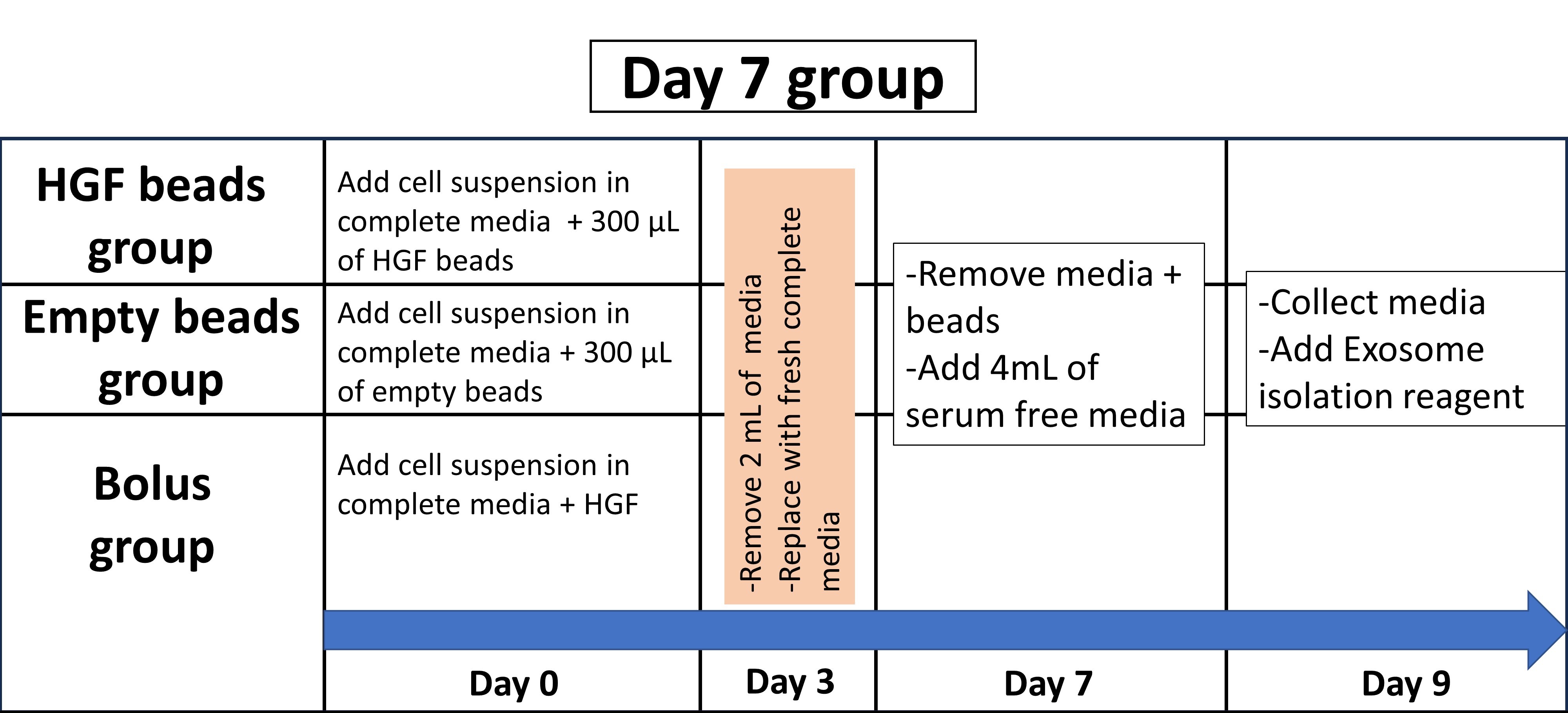

Supplement: Supplementary file 1 [file DataSheet1.zip › Figure S4 B.JPEG]

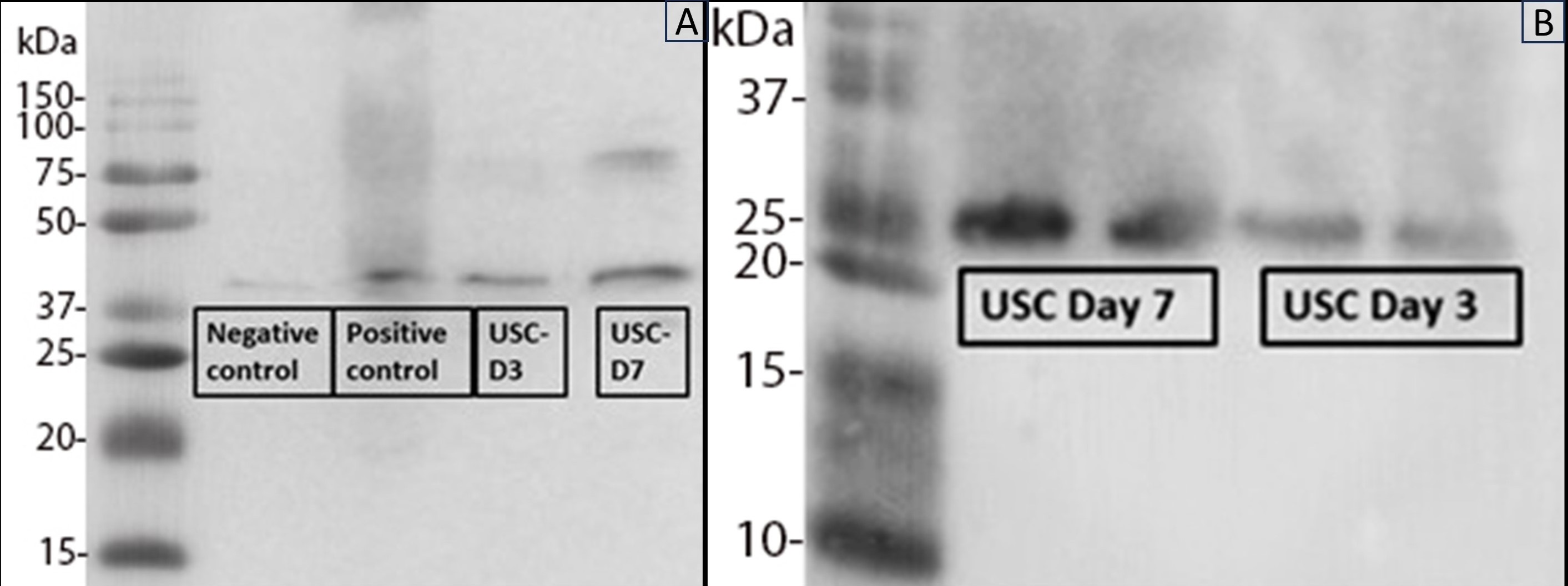

Supplement: Supplementary file 1 [file DataSheet1.zip › Figure S5.jpg]

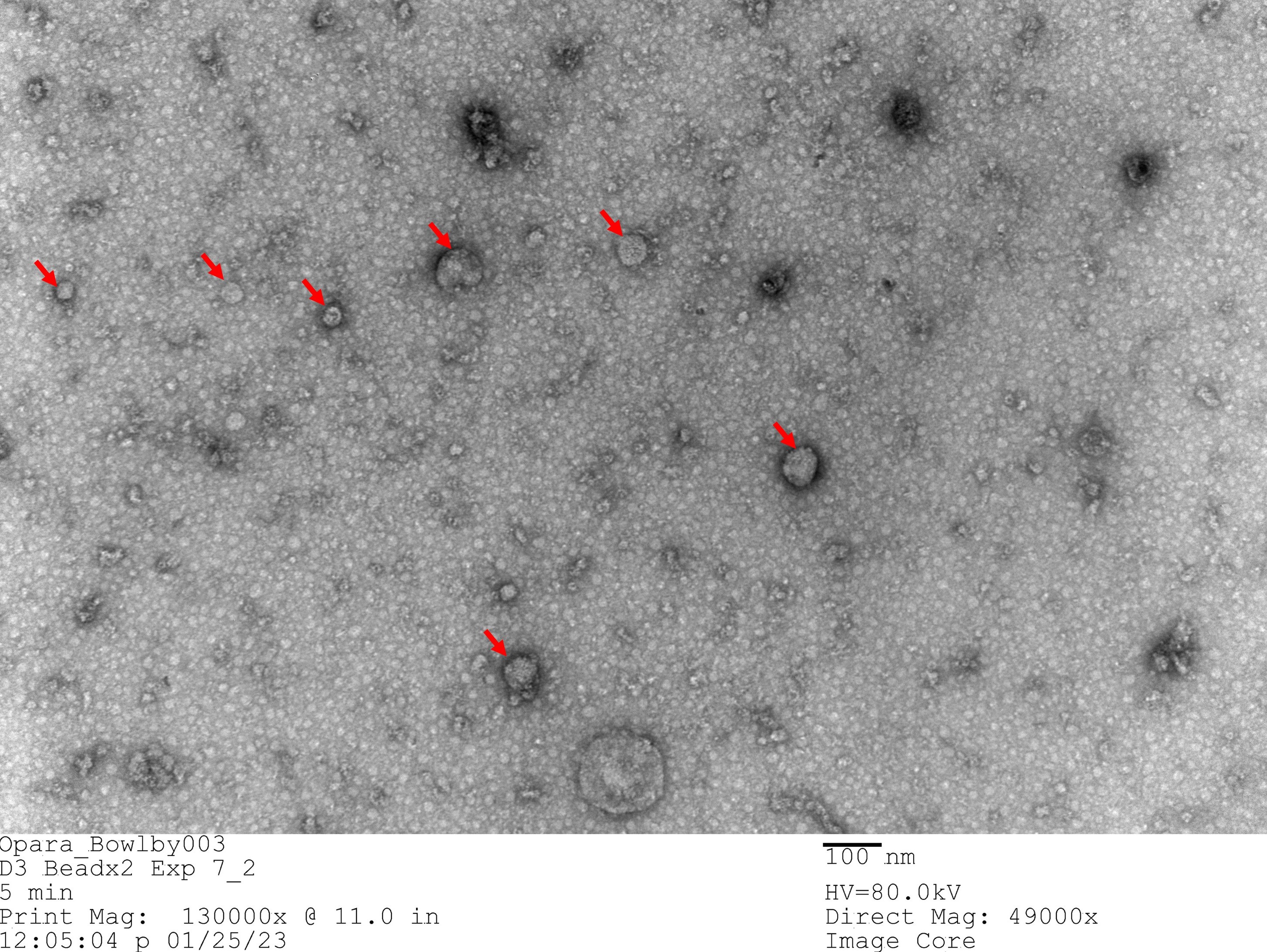

Supplement: Supplementary file 1 [file DataSheet1.zip › Figure S6.JPEG]
